# Identification of Central Regulatory Hubs in Pupal Diapause of *Helicoverpa armigera* Using Weighted Gene Co-Expression Network Analysis and Multiscale Embedded Network Analysis

**DOI:** 10.3390/insects17030352

**Published:** 2026-03-23

**Authors:** Zhe Song, Xinhui Liu, Jiawen Cao, Yujue Wang

**Affiliations:** School of Life Sciences, Zhengzhou University, Zhengzhou 450001, China

**Keywords:** Diapause, *Helicoverpa armigera*, transcriptomic analysis, co-expression network analysis

## Abstract

Diapause is a vital overwintering strategy for many insects, yet its comprehensive molecular architecture remains elusive. In the polyphagous pest *Helicoverpa armigera*, facultative pupal diapause is key to its ecological success. To elucidate the complex diapause regulatory network, we conducted a transcriptomic analysis of diapause (DP) versus non-diapause (NP) pupal brains across early pupal development (days 2, 5, and 10). Integrated analyses, including differential expression, persistent gene identification, weighted gene co-expression network analysis (WGCNA), and multiscale embedded network analysis (MEGENA), were employed to define core regulatory modules and hubs. The number of differentially expressed genes (DEGs) increased over time, with 1781 genes persistently regulated across all time points, enriched in mitochondrial metabolism, hormone signaling, and chromatin remodeling. WGCNA revealed a diapause-associated module (red) linked to RNA processing/transcription and a development-associated module (blue) enriched for translation and mitochondrial metabolism. Network analyses pinpointed three central hub genes: DDX5 and PLK4 (downregulated in diapause, upregulated upon 20E treatment) and TAF5L (upregulated in diapause, downregulated after 20E). This study provides a systems-level view of the transcriptional landscape governing pupal diapause in *H. armigera* and identifies novel candidate regulators for future functional studies.

## Simple Summary

Diapause is a programmed dormancy that helps insects survive unfavorable seasons. The cotton bollworm, *Helicoverpa armigera*, uses pupal diapause to overwinter, making it a widespread agricultural pest. Understanding how diapause is controlled at the molecular level could lead to new ways to manage this pest. In this study, we conducted a comprehensive, multi-faceted transcriptomic investigation, combining differential expression analysis, WGCNA, and MEGENA to progress from gene lists toward functional modules and central regulatory hubs. We found that diapause involves extensive changes in gene expression, especially in pathways related to IgSF CAM signaling and nucleocytoplasmic transport pathways, which have not been previously linked to insect diapause. Using gene network analysis, we identified three candidate genes, DDX5, PLK4, and TAF5L. These findings provide new insights into the genetic control of diapause and highlight potential targets for disrupting this process in pest populations.

## 1. Introduction

Diapause is a crucial adaptive strategy that enables insects to survive predictable periods of unfavorable environmental conditions like winter or drought [1,2,3,4]. It typically occurs at a specific developmental stage (e.g., embryonic, larval, pupal, and adult stages) and functions as a vital overwintering mechanism, characterized by a significant reduction in metabolic activity, enhanced stress tolerance, and the suspension of growth and reproduction [5,6,7]. The induction, maintenance, and termination of diapause are coordinated by complex interactions between environmental cues and endogenous hormonal and genetic programs [8,9,10,11]. For example, the typical lepidopteran pest *Helicoverpa armigera* detects short-day photoperiod and low-temperature signals during the autumn larval stage [12]. Subsequently, down-regulation of prothoracicotropic hormone (PTTH) in the brain leads to reduced production of the steroid hormone ecdysone in the prothoracic glands; low ecdysone levels prevent *H. armigera* from undergoing metamorphosis to the adult stage, thereby inducing pupal diapause as an adaptive strategy to survive cold winter conditions [13,14].

In Lepidoptera, pupal diapause is mainly regulated by the crosstalk among PTTH, juvenile hormone (JH), and ecdysteroids [1]. Suppression of ecdysteroid biosynthesis and signaling is a well-established hallmark of pupal diapause, and the essential role of ecdysteroids in diapause termination and developmental resumption has been clearly demonstrated [1,15]. However, the integrated temporal transcriptional dynamics and core gene regulatory networks underlying the initiation and maintenance of pupal diapause in *H. armigera* remain poorly and systematically characterized. Furthermore, given that *H. armigera* is a major polyphagous pest of global agricultural importance, deciphering the molecular basis of diapause in *H. armigera* is therefore of significant biological interest and may inform the development of novel, targeted pest management strategies.

Previous studies have characterized protein expression changes associated with diapause in *H. armigera*, revealing alterations in metabolic pathways, stress responses, and hormone signaling [5,16]. However, these investigations have largely focused on the identification of differentially expressed proteins, without integrating functional network-level analyses. Weighted Gene Co-expression Network Analysis (WGCNA) and Multiscale Embedded Gene Co-expression Network Analysis (MEGENA) represents sophisticated bioinformatic frameworks designed to elucidate complex regulatory relationships among genes, particularly in the context of high-throughput expression data [17,18]. Within entomological research, WGCNA has been successfully applied to identify genes involved in molting in *Leptinotarsa decemlineata* and to delineate key hormonal regulators in *Metisa plana* [19,20]. More recently, Liao et al. (2024) employed WGCNA to investigate diapause in *Pieris rapae*, revealing the critical role of the ecdysteroid response signaling pathway [21]. Collectively, these findings underscore the utility of co-expression network analysis as a robust approach for identifying candidate regulators of diapause and for systematically characterizing the regulatory networks underpinning this complex physiological process.

Given that the brain serves as the central regulatory hub for pupal diapause in *H. armigera*, it can perceive environmental cues and transduce them into hormonal signals, then orchestrate diapause-specific physiological responses in peripheral tissues throughout the body by secreting and regulating key hormonal signals [5,14]. We therefore prioritized pupal brains as the research subject in this study, aiming to decipher the core molecular regulatory network of pupal diapause. In the present study, we performed a comprehensive multi-omics transcriptomic analysis integrating differential expression profiling, WGCNA, and MEGENA to transition from the identification of differentially expressed genes to the elucidation of functional modules and central regulatory hubs. This integrated approach aims to establish a systems-level understanding of the regulatory architecture governing pupal diapause and to identify novel candidate genes that may play critical roles in this fundamental developmental process.

## 2. Materials and Methods

### 2.1. Data Acquisition and Preprocessing

(1) RNA Sequencing and Data Acquisition

Raw sequencing data for all samples were obtained as SRA files from the NCBI Sequence Read Archive (SRA) database under BioProject accession number PRJNA750814 (seen in Table 1). All diapause (DP) and non-diapause (NP) samples were collected and sequenced under identical experimental conditions.

(2) Transcriptomic Data Processing and Quantification

The raw SRA files were converted to FASTQ format using fasterq-dump from the SRA Toolkit (version: 3.2.1). Trimmomatic (version: 0.40) was then used to trim adapter sequences and low-quality reads with the following parameters: LEADING:3, TRAILING:3, SLIDINGWINDOW:4:15, MINLEN:36, and the prefix pair ‘TACACTCTTTCCCTACACGACGCTCTTCCGATCT’ and ‘GTGACTGGAGTTCAGACGTGTGCTCTTCCGATCT’ [22]. After quality control, reads were aligned and quantified at the transcript level using Salmon (version: 1.10.0) in quasi-mapping mode. A decoy-aware transcriptome index was constructed from the *H. armigera* reference transcriptome (assembly ASM3070526v1) and the genome sequence [23]. Transcript-level abundance estimates (TPM, Transcripts Per Kilobase of exon model per Million mapped reads) were aggregated to the gene level with the tximport R package (version: 1.38.1) using the corresponding GTF annotation file, which was downloaded from http://metazoa.ensembl.org (accessed on 7 August 2025), to produce an initial gene expression matrix.

(3) Data Filtering

Genes with low expression were then filtered out, retaining only those with TPM greater than 1 in at least three samples across the entire dataset. The sample-level and gene-level expression distribution after data clean are seen in Supplementary Figure S1.

### 2.2. Differential Expression Analysis

Differential gene expression between the DP and NP groups at days 2, 5, and 10 was analyzed using the limma R package [24]. Statistically significant genes were defined as those with an absolute log_2_ fold change (|log_2_FC|) > 0.5 and a Benjamini–Hochberg adjusted *p*-value (padj) < 0.05.

### 2.3. Functional Enrichment Analysis

Gene Ontology (GO) and Kyoto Encyclopedia of Genes and Genomes (KEGG) pathway enrichment analyses for the persistent differentially expressed genes (DEGs) were conducted using the clusterProfiler R package, with all expressed genes in the filtered matrix serving as the background gene set [25,26]. Terms exhibiting a fold enrichment > 1.5 and an adjusted *p*-value (padj) < 0.05 were deemed significantly enriched. Reactome pathway enrichment was performed using the online STRING tool (https://cn.string-db.org, version: 12.0, accessed on 26 July 2023), applying a false discovery rate (FDR) threshold of <0.05 and a required interaction score of >0.01.

### 2.4. Weighted Gene Co-Expression Network Analysis (WGCNA)

A co-expression network was constructed using the WGCNA R package [17]. Firstly, a union set encompassing all DEGs detected at the three time points (days 2, 5, and 10) was constructed as the initial input; subsequently, genes corresponding to the bottom 10% of variance values were filtered out to reduce noise interference. The soft-thresholding power (β) was selected as the smallest value that satisfied the criterion of scale-free topology fitting index (R^2^) ≥ 0.8, while ensuring a moderate mean connectivity and stable module partitioning for WGCNA. A topological overlap matrix (TOM) was calculated, and genes were hierarchically clustered using average linkage. Dynamic tree cutting was performed with the parameters deepSplit = 2 and minClusterSize = 30 to identify gene modules. Module-trait associations were calculated by correlating the module eigengene (first principal component of the module) with the diapause trait. To verify the robustness of the co-expression network and module preservation, two independent validations were performed: (1) TOM similarity verification: Pearson correlation coefficients were calculated between the topological overlap matrix (TOM) of the original dataset and resampled subsets, with a mean coefficient > 0.85 confirming the topological stability of the network. (2) Module preservation analysis: The module Preservation function in the WGCNA package was used for quantitative assessment with Z-summary scores as the core index; the diapause-associated red module and development-associated blue module both had Z-summary scores > 12, indicating strong module preservation. Hub genes within significant modules were defined as those with a |Gene Significance (GS)| > 0.5 and a |Module Membership (MM)| > 0.8.

### 2.5. Multiscale Network Analysis (MEGENA) and Protein–Protein Interaction (PPI)

For a more refined network analysis, we employed the MEGENA R package to identify multi-scale clusters from the top hub genes of the most diapause-relevant modules [18]. Core parameters were optimized: min.size was dynamically calculated as max (5, floor (vcount (network) × 0.05)) to avoid unstable small clusters while retaining biologically meaningful ones; mod.pval = 0.05 and hub.pval = 0.05 followed standard MEGENA thresholds; n.perm = 10 balanced efficiency and statistical power for small samples. Robustness was verified by two validations: (1) Spearman’s r > 0.9 for hub gene ranking across technical replicates confirmed reliable hub identification; (2) core diapause-associated clusters showed >85% Jaccard similarity in gene composition when re-running MEGENA with correlation thresholds 0.35–0.45, supporting stable cluster identification.

The top 25 genes ranked by hub.score within each module were selected for protein–protein interaction (PPI) network prediction. We submitted their protein sequences to the STRING database (https://cn.string-db.org, accessed on 26 July 2023) for the construction of PPI networks. Given that our study focuses on a non-model organism, we applied a medium confidence threshold from the STRING database to retain sufficient interactions while maintaining reliability. The resulting PPI networks were subsequently visualized and analyzed in Cytoscape (version: 3.10.4).

### 2.6. Candidate Gene Expression Analysis

(1) Animals

*H. armigera* larvae were reared on an artificial diet at 20 °C under two distinct photoperiod regimes: 14 h light/10 h dark for non-diapause-destined pupae, and 10 h light/14 h dark for diapause-destined pupae. Pupal brains (each biological replicate containing 12–15 pooled pupal brains) were dissected in ice-cold 0.75% NaCl saline and stored at −80 °C until subsequent experimental use.

(2) Injection of 20E to diapause pupae

20-Hydroxyecdysone (20E, Sigma–H5142, ≥93%(HPLC), St. Louis, MO, USA) was dissolved in ethanol to a final concentration of 0.67 μg/μL. A volume of 3 μL of this 20E solution was injected into day-20 diapause-destined pupae, with equal volume of pure ethanol serving as the control. Pupal brains were dissected at 24 h post-injection for subsequent analysis.

(3) Quantitative real-time PCR

Total RNA was extracted from pupal brains using 1 mL TRIzol^TM^ Reagent (Invitrogen, Yokohama, Japan) according to the manufacturer’s instructions. First-strand cDNA was synthesized as PCR template through an M-MLV reverse transcription system (Promega, Madison, WI, USA). Quantitative real-time PCR was performed with specific primers (Supplementary Table S1) on a light Cycler480 (Roche, Rotkreuz, Switzerland) using 2×SYBR Premix Ex Taq (Takara, Beijing, China). The expression levels of actin in *H. armigera* were used as an internal control. The qPCR data were analyzed using the 2^−ΔΔCT^ method as described previously [27].

(4) Statistical analysis

Expression profiles of these genes across developmental time in DP and NP brains, and in response to 20E treatment, were plotted and statistically assessed with GraphPad 8.0.1. Quantitative data were presented as bar graphs, with values shown as the mean ± standard deviation (SD) of three biological replicates (two technical replicates in each biological replicate). Paired Student’s *t*-tests were used to detect significant differences for the expression of candidate genes in the brains of NP and DP pupae across days 2, 5, and 10, and the effects of 20E injections. In cases where multiple *T*-tests were run, a Bonferroni correction was applied to adjust the *p*-value. Statistical significance is denoted as * for *p* < 0.05 and ** for *p* < 0.01.

## 3. Results

### 3.1. Differential Gene Expression Analysis

**Figure 1:**
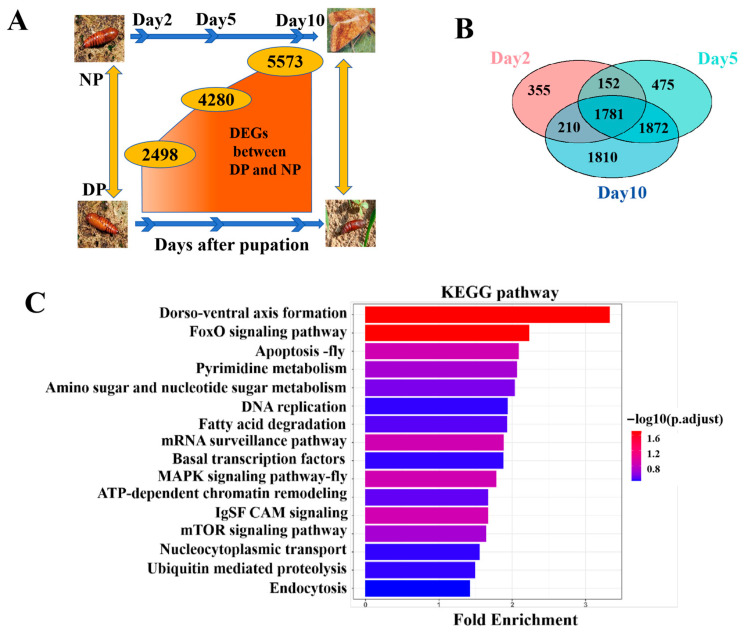
Differential Gene Expression Analysis between NP and DP from day 2 to day 10. (**A**) DEGs between NP and DP at 2, 5, 10 days after pupation. (**B**) Venn diagram analysis of DEGs across different days. (**C**) KEGG pathway analysis of intersection of day 2, 5, and 10 DEGs.

Nondiapause pupae (NP) develop into adults within 23–24 days, whereas diapause-destined pupae (DP) enter diapause approximately 8–10 days post-pupation, with a pupal lifespan exceeding 3 months. Consequently, we focused on the diapause initiation period, specifically from day 0 to day 10 of pupal development. Differential expression between DP and NP pupal brains at days 2, 5, and 10 was analyzed, and the results showed that the number of DEGs increased over time (Figure 1A), indicating that transcriptional divergence between diapause and developmental states amplifies as pupal development progresses.

As shown in Figure 1B, a total of 1781 genes were consistently differentially expressed across all three time points (intersection of day 2, 5, and 10 DEGs). GO enrichment analysis revealed that these persistent DEGs were significantly involved in mitochondrial metabolism, hormone metabolic processes, protein phosphorylation, and chromosome organization (Figure S2). KEGG pathway analysis further indicated enrichment in multiple signaling and metabolic pathways, including FoxO signaling pathway, dorso-ventral axis formation, IgSF CAM signaling, apoptosis, MAPK signaling pathway, mRNA surveillance pathway, mTOR signaling pathway, pyrimidine metabolism, amino sugar and nucleotide sugar metabolism, ATP-dependent chromatin remodeling, fatty acid degradation, DNA replication, nucleocytoplasmic transport, ubiquitin-mediated proteolysis, basal transcription factors, and endocytosis (Figure 1C).

### 3.2. Weighted Gene Co-Expression Network Analysis

**Figure 2:**
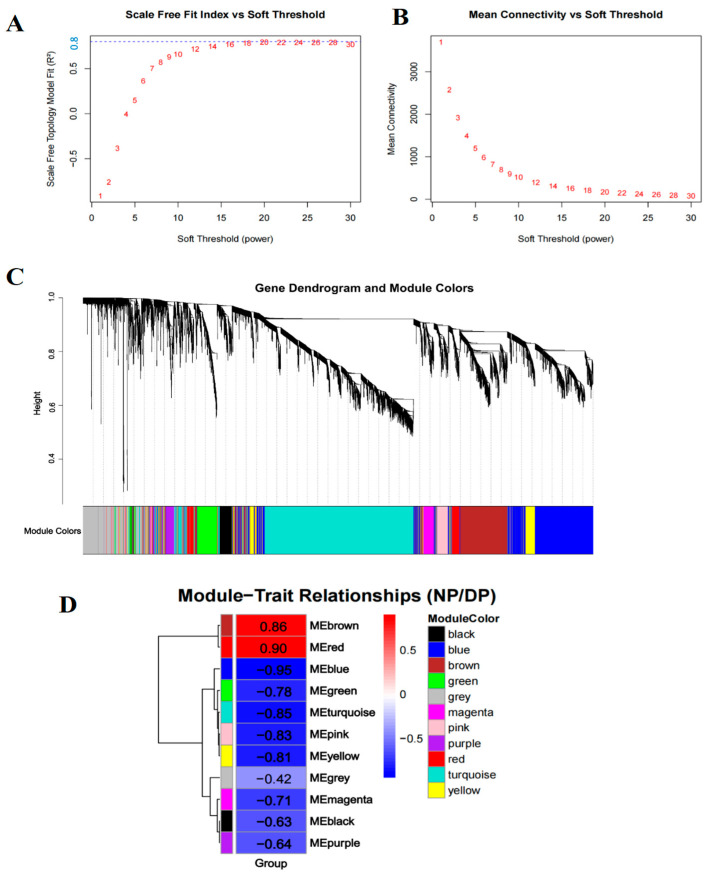
Co-expression analysis for all differentially expressed genes at three time points. (**A**,**B**) The scale-free fit index and mean connectivity of WGCNA. (**C**) The gene dendrogram and module colors. (**D**) The module-trait relationships. Red fill color indicates a positive correlation with diapause, while blue fill color represents a negative correlation.

To identify key regulatory genes associated with diapause in *H. armigera*, we performed WGCNA on the union set of DEGs from days 2, 5, and 10, followed by exclusion of the 10% of genes with the lowest variance. Sample clustering based on gene expression profiles corresponded clearly to diapause and developmental phenotypes, confirming that transcriptomic variation underlies the physiological divergence between these states (Figure S3). A soft-thresholding power of 20 was selected (scale-free topology fit index R^2^ = 0.8; mean connectivity = 172) to construct a signed co-expression network (Figure 2A,B). Using the parameters deepSplit = 2 and minClusterSize = 30, 11 distinct modules were identified (Figure 2C). Module–trait correlation analysis revealed that the red module exhibited the highest positive correlation with diapause, whereas the blue module showed the strongest negative correlation (i.e., positive correlation with development) (Figure 2D). These two modules were selected for further investigation.

**Figure 3:**
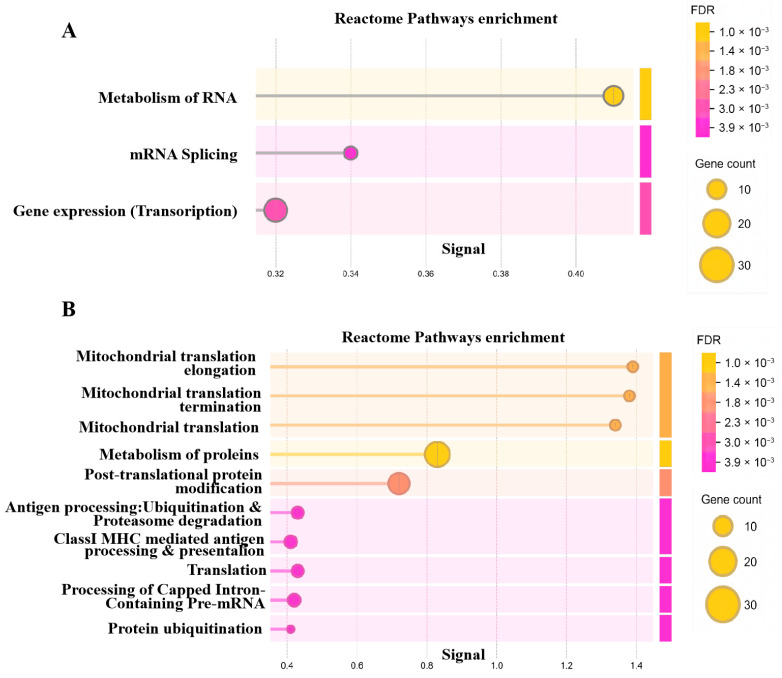
The reactome pathways enrichment of hub genes in red (**A**) and (**B**) blue modules.

Within each module, genes with |GS| > 0.5 and |MM| > 0.8 were defined as hub genes highly associated with the respective trait. Reactome pathway enrichment analysis of these hub genes demonstrated that genes in the diapause-related red module were primarily involved in RNA metabolism, transcription, and splicing (Figure 3A). In contrast, genes in the development-associated blue module were enriched for mitochondrial metabolism, protein metabolism, translation, and protein ubiquitination and degradation (Figure 3B).

### 3.3. MEGENA Network and Protein–Protein Interaction (PPI) Analysis

**Figure 4:**
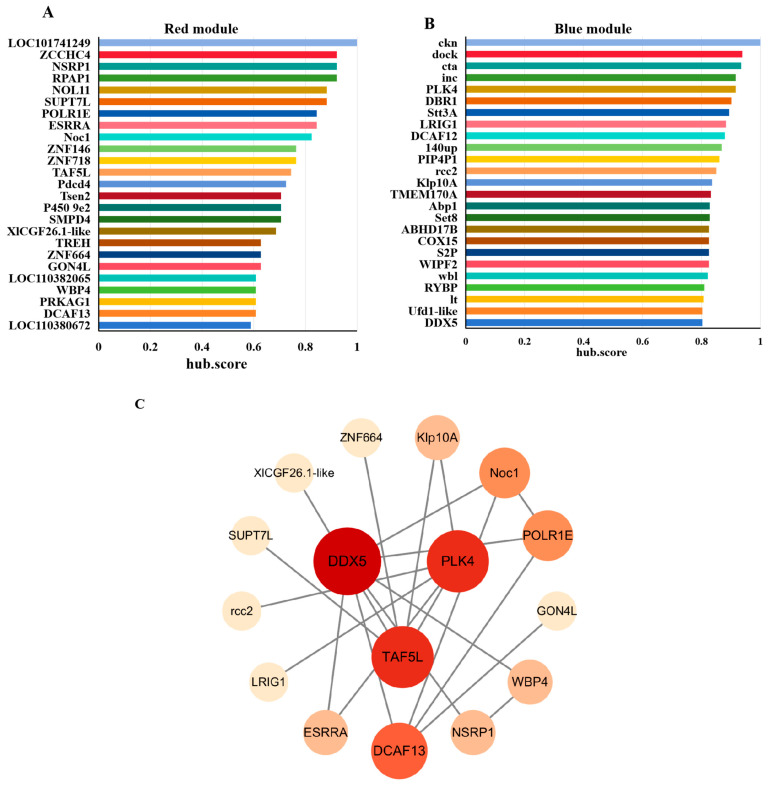
Screening of hub genes. The TOP25 hub genes in red (**A**) and blue (**B**) modules ranked by hub.score from MEGENA Network. (**C**) PPI Analysis of hub genes. The darker of fill color and the larger of node size represent the higher connectivity of genes.

To further refine key regulators, we applied MEGENA (Multiscale Embedded Gene Co-expression Network Analysis) to the significant genes from the red and blue modules. The top 25 hub genes ranked by hub.score from each module were selected (Figure 4A,B). Their amino acid sequences were submitted to the STRING database to predict protein–protein interactions, and the resulting PPI networks were visualized using Cytoscape (Figure 4C). Three genes including DDX5 (DEAD-box helicase 5), PLK4 (Polo-like kinase 4), and TAF5L (TATA-box binding protein associated factor 5-like) occupied central positions within the PPI networks, suggesting their potential roles in regulating diapause in *H. armigera*.

### 3.4. Expression Dynamics of DDX5, PLK4, and TAF5L in the Brains of NP and DP Pupae from Day 2 to Day 10

**Figure 5:**
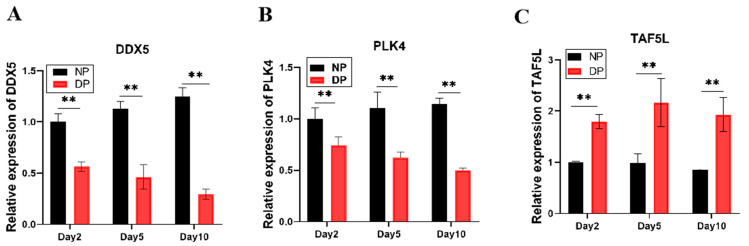
(**A**) Expression Dynamics of DDX5 in the brains of NP and DP pupae from day 2 to day 10; (**B**) Expression Dynamics of PLK4 from day 2 to day 10; (**C**) Expression Dynamics of TAF5L in the brains of NP and DP pupae from day 2 to day 10. ** indicates *p* < 0.01 (NP vs. DP, determined by multiple *t*-test with Bonferroni correction).

To validate these hub genes, we examined the relative expression patterns of DDX5, PLK4, and TAF5L in the brains of NP and DP pupae across days 2, 5, and 10 (Figure 5). DDX5 expression increased over time in developing pupae but declined in diapause-destined pupae, with significantly higher levels in NP than in DP brains. Similarly, PLK4 expression was markedly elevated in NP compared to DP brains at all time points. In contrast, TAF5L expression was substantially higher in diapause-destined pupal brains relative to developing ones. These results indicate that DDX5 and PLK4 are negatively correlated with diapause, whereas TAF5L is positively correlated with diapause, consistent with the WGCNA module assignments.

### 3.5. Expression Changes in DDX5, PLK4, and TAF5L upon Diapause Termination

**Figure 6:**
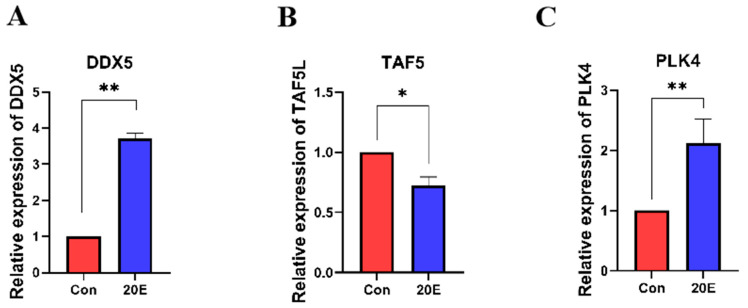
(**A**) Expression Changes in DDX5 upon Diapause Termination; (**B**) Expression Changes in PLK4 upon Diapause Termination; (**C**) Expression Changes in TAF5L upon Diapause Termination. Injection with DMSO was set as a control (Con). * indicates *p* < 0.05 and ** indicates *p* < 0.01 (determined by a paired Student’s *t*-test).

Ecdysteroids, particularly 20-hydroxyecdysone (20E), are known to trigger the transition from diapause to development in insects [28]. To investigate the expression changes in DDX5, PLK4, and TAF5L in response to ecdysteroids, we detected and compared the expression levels of these target genes between 20E-treated and DMSO-treated diapause pupae. Following 20E administration, DDX5 and PLK4 were significantly up-regulated as compared to DMSO treatment, while TAF5L expression was markedly down-regulated in formerly diapausing pupae (Figure 6). These shifts align with the expression patterns observed during natural development and further support the functional importance of DDX5, PLK4, and TAF5L in the molecular switch between diapause and development in *H. armigera*.

## 4. Discussion

Insect diapause involves significant physiological and developmental reprogramming, but a systematic and comprehensive transcriptional architecture has not been established. We constructed a time-resolved transcriptomic atlas of diapause and development in *H. armigera* pupal brains by integrating differential expression analysis, persistent regulatory signatures, and systems-level network analyses. This integrated approach confirms established regulatory paradigms while also uncovering the layered complexity of gene regulation that regulates pupal diapause or development.

Differential gene expression analysis revealed a crucial temporal dimension to diapause, as the transcriptional divergence between diapause and non-diapause pupal brains from 2 to 10 days post-pupation. The increase from 2498 to 5573 differentially expressed genes indicates that diapause is not a static state but a dynamically established and actively maintained physiological condition. The identification of 1781 genes consistently differentially expressed across all three time points defines a core set of regulatory and effector genes indispensable for diapause. Functional enrichment of this persistent gene set was highly informative. Its strong association with mitochondrial metabolism and ATP-dependent chromatin remodeling aligns with the hallmark metabolic depression of diapause, where energy production is finely tuned to match reduced maintenance demands while avoiding lethal depletion [1,29,30,31]. This observation is consistent with prior findings that key energy metabolism pathways, such as glycolysis and the TCA cycle, exhibit higher activity in developing pupal brains, and their inhibition can significantly arrest development [29,32,33,34]. Concurrent enrichment in hormone metabolism pathways also underscores the central role of hormonal regulation. In addition, the enrichment of terms like protein phosphorylation and chromosome assembly, along with pathways such as FoxO, mTOR, and MAPK signaling, reveals important signaling and cell cycle adjustments. Phosphorylation events mediated by key kinases (e.g., Akt, AMPK, MAPKs) are widely reported as pivotal in diapause regulation across species [34,35,36,37]. The FoxO signaling pathway, a conserved regulator of lifespan and stress resistance, is frequently implicated in diapause for its potential role in coordinating metabolic shift and stress response [38]. The downregulation of mTOR signaling, a master regulator of anabolism and cell growth, provides a plausible mechanistic link to the global suppression of biosynthesis and cell proliferation in diapause [37].

Most importantly, our study is the first to report the enrichment of IgSF CAM signaling and nucleocytoplasmic transport pathways in *H. armigera* pupal diapause; these pathways have not been previously associated with pupal diapause. IgSF CAMs are key mediators of cell–cell adhesion, intercellular communication, and signal transduction, particularly in the nervous system [39]. They regulate neural development, synaptic plasticity, and the transmission of extracellular signals into intracellular regulatory cascades. It has been reported that a critical affinity window for IgSF proteins DIP-α and Dpr10 is required for proper motor neuron arborization in *Drosophila* [40]. Nucleocytoplasmic transport governs the selective shuttling of proteins, RNAs, and signaling molecules between the nucleus and cytoplasm [41,42]. It is essential for transcriptional regulation, signal transduction, cell cycle control, and stress responses. Thus, IgSF CAM signaling and nucleocytoplasmic transport pathways may play roles in mediating brain-peripheral tissue communication and gene expression regulation during diapause. Collectively, these findings extend beyond the confirmation of known pathways and define new molecular signatures of *H. armigera* pupal diapause.

WGCNA and subsequent MEGENA/PPI analyses distilled global transcriptional changes into functional modules and identified central regulatory hubs [20]. The clear phenotypic separation of samples and the identification of distinct modules, a diapause-correlated red module enriched for RNA processing and transcription, and a development-correlated “blue” module for translation and mitochondrial metabolism, indicate a fundamental reorganization of cellular priorities. Our multi-step analysis converged on three genes, DDX5, PLK4, and TAF5L, as central nodes within these diapause-related co-expression networks. Their opposing expression trajectories during diapause maintenance and swift reversal upon 20E treatment imply they are candidate regulators in diapause.

DDX5 is an RNA helicase involved in multiple aspects of RNA metabolism, including transcription, splicing, ribosome biogenesis, and miRNA processing, and is a key facilitator of global gene expression and cellular proliferation [43,44,45]. PLK4 is a serine/threonine kinase that acts as the master regulator of centriole duplication, thereby controlling cell cycle progression, genome stability, and mitotic fidelity [46,47]. In our study, the sustained up-regulation of DDX5 and PLK4 in developing pupal brains aligns with their roles in active cellular biosynthesis and proliferation. Their suppression during diapause supports a model of globally suppressed transcription, translation, and cell division. Their position within the pro-development blue module underscores their potential role as downstream effectors executing the metabolic and biosynthetic reprogramming mandated by developmental signals.

TAF5L is a core component of a histone acetyltransferase complex crucial for chromatin remodeling and the initiation of RNA polymerase II-dependent transcription, playing a vital role in regulating specific gene sets [48]. In contrast to DDX5 and PLK4, TAF5L was significantly up-regulated in diapausing brains and sharply down-regulated after 20E treatment. This pattern suggests a potential role in actively establishing or maintaining a diapause-specific transcriptional program, possibly by modulating chromatin accessibility at key developmental gene loci. Its presence in the diapause-associated red module further supports a function in regulating the unique transcriptional landscape of diapause. Further investigation into the specific target genes of TAF5L in the diapause brain is warranted, as these may reveal critical pathways actively repressed for the maintenance of the dormant state.

This study elucidates the core transcriptional regulatory network and candidate hub genes underlying pupal diapause in *H. armigera*, yet several inherent limitations should be acknowledged. Owing to the limited sample size, we failed to construct a multi-trait WGCNA network integrating diapause status and temporal gradient, and an unusually high soft-threshold (β = 20) was adopted; instead, a single co-expression network based on preselected DEGs was constructed for overall analysis, which we recognize is insufficient. Our analysis was restricted to pupal brain tissue, failing to reflect the multi-tissue coordination and brain–peripheral tissue signaling crosstalk essential for systemic diapause regulation; this study relies solely on transcriptomic data without multi-omics validation, and transcriptional changes cannot be directly linked to protein abundance or metabolic flux. Furthermore, hormonal inferences were based only on hormone-related gene expression rather than direct titer quantification, and the three time points lacked sufficient resolution to capture fine-scale dynamic molecular changes throughout diapause. To address these limitations, future research will conduct integrated multi-tissue transcriptomic and metabolomic analyses, combine transcriptomics with proteomics for multi-omics validation, perform targeted hormone quantification and high-resolution time-series sampling, and functionally verify these candidate hub genes (DDX5, PLK4, TAF5L) via CRISPR/Cas9 and RNAi to clarify their biological roles in diapause regulation.

## 5. Conclusions

In summary, our integrated analysis delineates a systems-level view of the transcriptional landscape governing pupal diapause in *H. armigera*. We reveal a core set of persistently regulated genes and delineate co-expression networks that organize the diapause phenotype. Furthermore, we identify DDX5 and PLK4 as novel candidate regulators for developmental competence, whereas TAF5L is a potential mediator of diapause.

## Figures and Tables

**Table 1:** Raw sequencing data from non-diapause (NP) and diapause (DP) pupae.

Study Accession	Experiment Accession	Sample Accession	Run	Type	Time (Day)	Repeat
SRP330785	SRX11624942	SRS9659591	SRR15320453	NP	2 d	1
SRP330785	SRX11624941	SRS9659590	SRR15320454	DP	10 d	1
SRP330785	SRX11624940	SRS9659589	SRR15320455	DP	5 d	1
SRP330785	SRX11624939	SRS9659588	SRR15320456	DP	2 d	1
SRP330785	SRX11624938	SRS9659587	SRR15320457	NP	10 d	1
SRP330785	SRX11624937	SRS9659586	SRR15320458	NP	5 d	1
SRP330785	SRX11624936	SRS9659585	SRR15320459	NP	2 d	2
SRP330785	SRX11624935	SRS9659584	SRR15320460	DP	10 d	2
SRP330785	SRX11624934	SRS9659583	SRR15320461	NP	10 d	2
SRP330785	SRX11624933	SRS9659582	SRR15320462	NP	5 d	2
SRP330785	SRX11624932	SRS9659581	SRR15320463	NP	2 d	3
SRP330785	SRX11624931	SRS9659580	SRR15320464	DP	10 d	3
SRP330785	SRX11624930	SRS9659579	SRR15320465	DP	5 d	2
SRP330785	SRX11624929	SRS9659578	SRR15320466	DP	2 d	2
SRP330785	SRX11624928	SRS9659577	SRR15320467	NP	10 d	3
SRP330785	SRX11624927	SRS9659576	SRR15320468	NP	5 d	3
SRP330785	SRX11624926	SRS9659575	SRR15320469	DP	5 d	3
SRP330785	SRX11624925	SRS9659574	SRR15320470	DP	2 d	3

## Data Availability

The original contributions presented in this study are included in the article/Supplementary Materials. Further inquiries can be directed to the corresponding authors.

## Supplementary Materials

The following supporting information can be downloaded at https://www.mdpi.com/article/10.3390/insects17030352/s1: Figure S1: The sample-level and gene-level expression distribution after data clean; Figure S2: GO enrichment analysis of the consistently differentially expressed genes across all three time points; Figure S3: The sample clustering heatmap with traits; Table S1: Primers used in this work.

## Abbreviations

The following abbreviations are used in this manuscript:

DDX5	DEAD-box helicase 5
PLK4	Polo-like kinase 4
TAF5L	TATA-box binding protein associated factor 5-like
WGCNA	Weighted gene co-expression network analysis
MEGENA	Multiscale embedded network analysis
DP	Diapause pupae
NP	Non-diapause pupae
