# Supplementary material for: Identification of Central Regulatory Hubs in Pupal Diapause of Helicoverpa armigera Using Weighted Gene Co-Expression Network Analysis and Multiscale Embedded Network Analysis
Source: Insects. 2026 Mar 23;17(3):352. doi: 10.3390/insects17030352 (PMC13026625; doi:10.3390/insects17030352)
Supplement: Supplementary file 1 [file insects-17-00352-s001.zip › insects-4139021-supplementary.pdf]

Figure S1

A

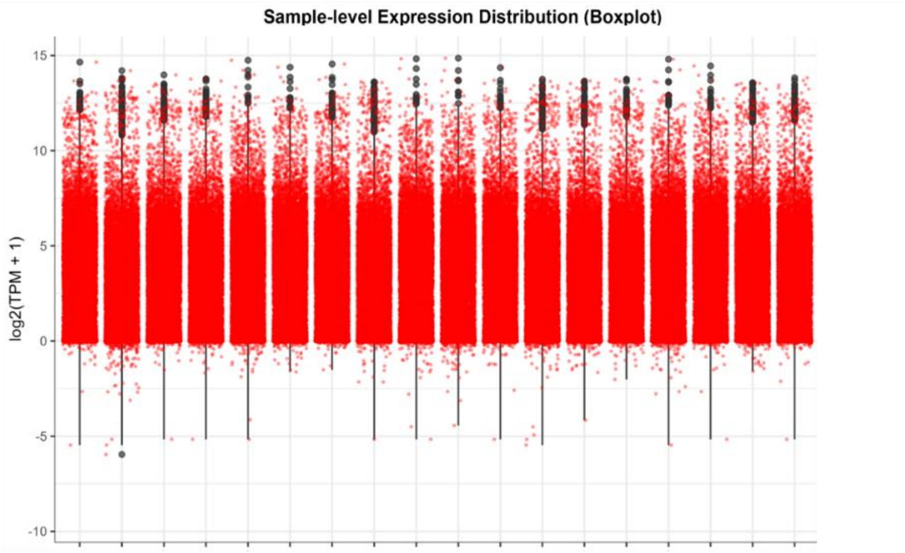

B

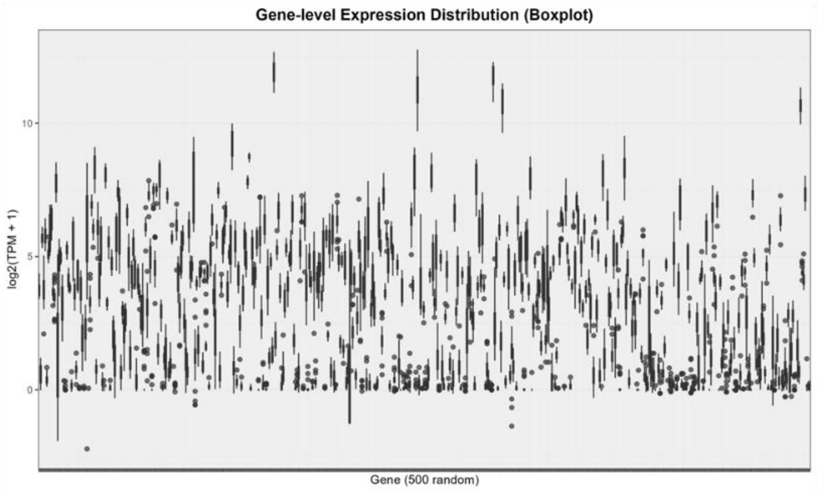

Figure S1 The sample-level (A) and gene-level (B) expression distribution after data clean.

Figure S2

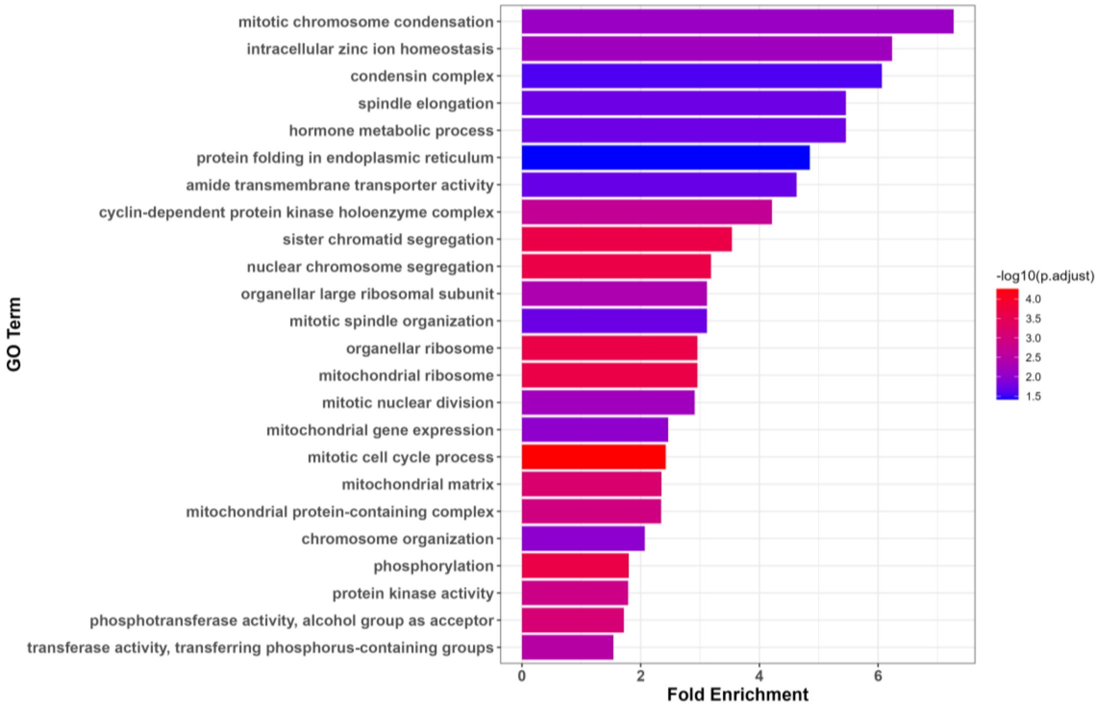

Figure S2 GO enrichment analysis of the consistently differentially expressed genes across all three time points

Figure S3

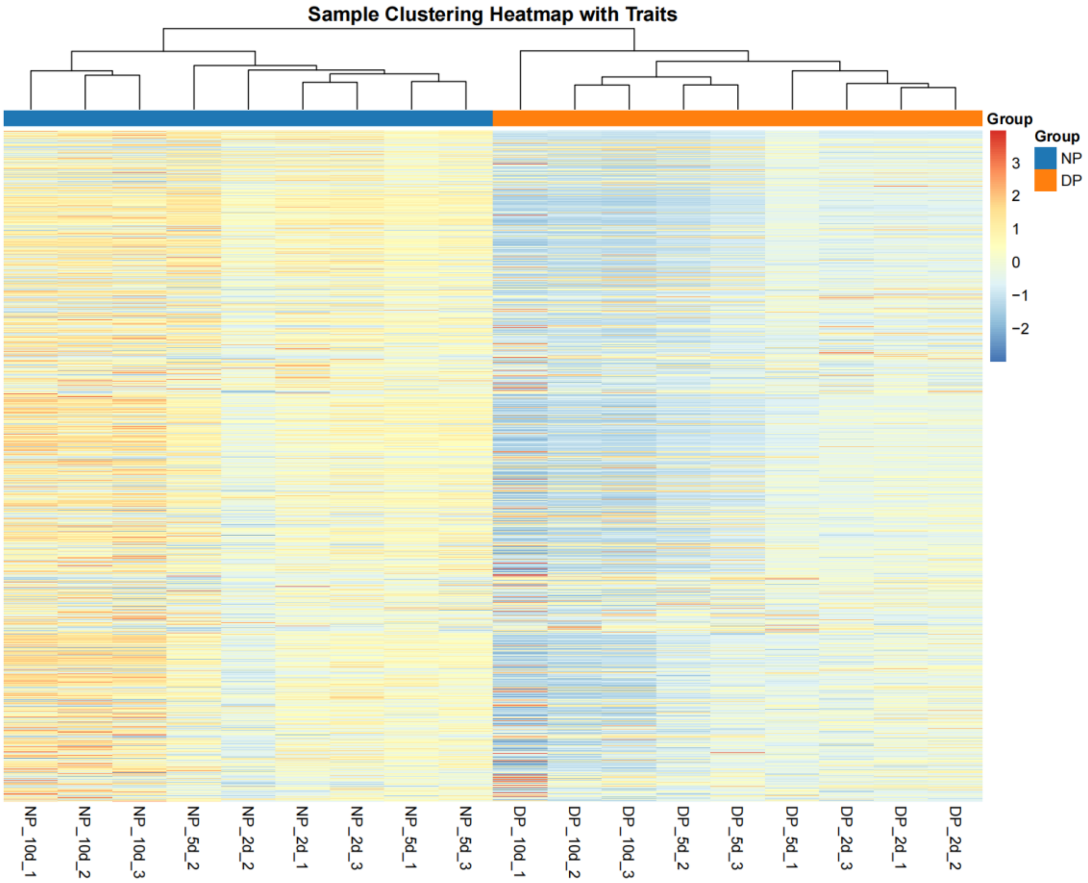

Figure S3 The sample clustering heatmap with traits.

Table.S1

Table.S1 Primers used in this work

TAF5L -F	5'-GGTAAAAAATGAGCTTCTTGGATT-3'
TAF5L -R	5'-GATTGCCGCTTTCAGGTCCTGTAA-3'
PLK4-F	5'-GAAGATGCACATTATGTTTATTTAG-3'
PLK4-R	5'-TCCATTGTACAAGAAGGGATGTTCT-3'
DDX5-F	5'-GAAGAAGGAGGGTTTCCAGATTA-3'
DDX5-R	5'-GCCAGTGGTGTGGCGATGACTAT-3'
Actin-F:	5’-CAACGGCATCCACGAGACCAC-3’
Actin-R:	5’-GGGCTGTGATCTCCTTCTGC-3’
